# The dual impact of education and occupation on cognitive functioning in older Mexican adults: A cross-sectional exploratory study

**DOI:** 10.1016/j.ssmph.2024.101738

**Published:** 2024-12-13

**Authors:** José Eduardo Cabrero Castro, Mariela Gutierrez, Theresa Andrasfay, Emma Aguila, Brian Downer

**Affiliations:** a Department of Population Health & Health Disparities, The University of Texas Medical Branch at Galveston, 301 University Boulevard, Galveston, TX, USA; b School of Public and Population Health, The University of Texas Medical Branch at Galveston, 301 University Boulevard, Galveston, TX, USA; c Department of Public Health, California State University San Marcos, 333 S Twin Oaks Valley Rd, San Marcos, CA, USA; d Sol Price School of Public of Policy, University of Southern California, Los Angeles, CA, 90007, USA

**Keywords:** Cognitive function, Educational attainment, Occupation

## Abstract

This research investigated the relationship between cognitive performance and an individual's educational attainment as well as occupational mental demands among Mexican adults aged 50 or older. We hypothesized that cognitively demanding work boosts cognitive performance for older adults regardless of their education level. To test our hypothesis, we analyzed data on 12,939 individuals in the 2012 Mexican Health and Aging Study using a Generalized Linear Model with a Gaussian family and identity link function. We assessed cognitive demands of occupations with the National Information Network's descriptors, focusing on worker-oriented and job-oriented mental demands. We found that greater worker-oriented (β = 0.5; CI = 0.45, 0.55) and job-oriented (β = 0.49; CI = 0.45, 0.53) mental demands predicted better cognitive performance. Educational attainment correlated even more strongly with better cognitive performance (β = 0.9; CI = 0.87, 0.92). Both our models showed a statistically significant negative interaction between medium occupational mental demands and medium education level (job-oriented: β = −0.09; CI = −0.14, −0.05; worker-oriented: β = −0.07; CI = −0.12, −0.02). Other interaction terms were not significant. This study highlighted a significant effect of educational attainment on cognitive function, which is more pronounced than that of occupational mental demands. The association of higher occupational mental demands with higher cognitive function appeared to be largely independent of educational background. The similarity in cognitive scores using worker-oriented or job-oriented metrics suggests that both are useful for assessing occupational mental demands. Education and cognitive engagement at work are crucial for promoting cognitive health in aging populations.

## Highlights

• Cognitively complex occupations lead to better cognitive scores.
• The effect of occupational mental demands appears to be independent of education.
• Association of education with cognitive function is stronger than that of occupation.
• O∗NET metrics effectively assess the relation of job complexity and cognition.

## 1 Introduction

Like other countries in Latin America, Mexico is rapidly aging. Population aging in Mexico will likely contribute to an increase in the prevalence and incidence of cognitive impairment and dementia(M. Prince et al., 2012; Wong & Palloni, 2009). Estimates for the prevalence of dementia among Mexicans 65 or older range from 5.4% to 8.4%(M. Prince et al., 2008, Prince et al., 2013; M. J. Prince, de Rodriguez, et al., 2008). Demographic projections indicate that the proportion of individuals 60 or older in Mexico will increase from 3.9% in 2000 to 16.1% by 2050(CONAPO, 2004). This context underscores the need for targeted healthcare strategies and a deeper understanding of the factors associated with cognitive aging, particularly the social and economic factors that have been less studied among the Mexican population.

Previous research has explored the influence of education on late-life cognitive function among older adults in Mexico. Specifically, formal education is associated with decreased odds of cognitive impairment relative to those with no formal education among Mexican adults aged 50 or older(Gonçalves et al., 2023). Furthermore, higher educational attainment is linked to a lengthier cognitively healthy life expectancy(Cabrero-Castro et al., 2023). By many measures, Mexican education and literacy has improved in the past century. In 1930, 66.8% of the population over the age of 15 was illiterate(Cruz et al., 2015). Average years of schooling reached 2.8 years in 1960 and 3.6 years in 1970(Medellín & Izquierdo, 2012). By 2010, the illiteracy rate in Mexico had decreased to 8%, an improvement largely attributed to public policies implemented in the 1980s, which enabled 35% of the population aged 13 to 18 to receive post-primary education, and 11% of the population aged 18 to 25 to attain higher education(Cruz et al., 2015). Research using data from the Mexican Health and Aging Study (MHAS) has suggested that more recent cohorts exhibit better cognitive health than their older counterparts(Díaz-Venegas et al., 2019), likely due to these educational reforms and greater educational attainment(Creighton & Park, 2010).

Previous research has seldom studied occupation as a determinant of aging outcomes among the Mexican population. The relationship between occupation and cognitive health is well established in high-income countries. Intellectual stimulation, which can occur in the occupational environment, is associated with lower risk for cognitive decline and dementia(Finkel et al., 2009; Fujishiro et al., 2019a; Smart, 2015). Complex work environments may build cognitive reserve, increasing an individual's ability to compensate for age- or disease-related changes in the brain and to preserve function as well as to prevent or delay cognitive decline(Baldivia et al., 2008; Chapko et al., 2018). Cognitive reserve is associated with preserved global cognitive function and memory domains in late-life, and the accumulation of cognitive reserve abilities are important in preventing cognitive decline(X. Li et al., 2021). Both educational attainment and occupational mental demands are life course determinants of cognitive reserve(Chapko et al., 2018).

There has been limited research in Mexico addressing how occupational demands influence cognition among older adults, despite the country's shift toward industrialization resulting in a transition from low-skill to more complex middle-skill jobs(Gutiérrez, 2012). In 1930, 71.7% of the economically active population was employed in agriculture and livestock activities. Eighty years later, 62.3% of the population was working in the tertiary economic sector, primarily represented by manufacturing, commerce, and activities related to the real estate subsector(Cruz et al., 2015). In addition, the percentage of economically active women increased from 7.2% in 1930 to 17% in the 1970s and 33.3% in 2010(García et al., 2001).

The changes in Mexican occupations over recent decades suggest the need for a life course approach to understand the socioeconomic contributors to cognitive health in Mexico(Downer et al., 2022; Zeki Al Hazzouri et al., 2011). Health trajectories are the cumulative product of risks and protective factors. The link between education and occupation can be conceptualized through a chain of risk model, characterized by a succession of interconnected exposures that exert influence on disease risk(Ben-Shlomo & Kuh, 2002; Kuh, 2003). This construct elucidates the effects of early life experiences and subsequent adult exposures as chain reactions. It posits that each discrete exposure not only escalates the risk of subsequent exposures but also exerts an independent effect on disease susceptibility—a phenomenon denoted as an "additive effect."(Kuh, 2003) This chain of risk and additive effect model is the framework through which we can explain how education and occupation together and separately shape trajectories across the life course.

The additive effects of education and occupation on cognitive function have been extensively investigated in high-income populations but not in low- and middle-income countries. For example, a study of the relationship between mental work demands and cognitive performance among older adults in Germany demonstrated a significant association between greater mental work demands and enhanced cognitive function(Then et al., 2014). Intriguingly, this effect was evident for persons of all education levels, and no interaction effects were observed. This suggests that high mental work demands contribute independently to cognitive functioning. No research to date has explored this additive relationship in Mexico. There is preliminary evidence suggesting such additive effects may occur. A recent study in Mexico, for example, reported higher wage returns resulting from additional schooling achieved through occupational advancement(Pearlman & Rubb, 2020). Therefore, it remains an open question whether the additive effects of education and occupation on cognitive function manifest among older adults in Mexico. This question is particularly pertinent given the country's increase in educational attainment over the past century, as well as its transformation from a predominantly agriculture-based economy to a service-oriented one that also maintains significant activity in mining and construction. These changes provide a rich context for examining the interplay between occupational profiles, varying degrees of education, and cognitive outcomes.

This study examines how education and main lifetime occupation relate to cognitive functioning. It explores a potential additive effect of education and occupation on cognition among Mexican adults aged 50 or older. We hypothesize that higher mental demands at work are positively associated with better performance in cognitive tests, independent of an individual's level of education. We further hypothesize that there are additive effects of high mental demands at work, indicating that such demands contribute to cognitive functioning beyond the influence of education.

## 2 Methods

### 2.1 Mexican Health and Aging Study

We drew our data from the Mexican Health and Aging Study (MHAS). The MHAS is an ongoing, population-based cohort study focused on population aging in Mexico(Wong et al., 2017). It began in 2001 with a nationally representative sample of 15,186 participants aged 50 or older, including spouses or partners living in the same household regardless of age. Follow-up interviews occurred in 2003, 2012, 2015, 2018, and 2021. New participants and their spouses were added in the 2012 and 2018 waves to ensure continued representation of the population in the survey.

**Fig. 1:**
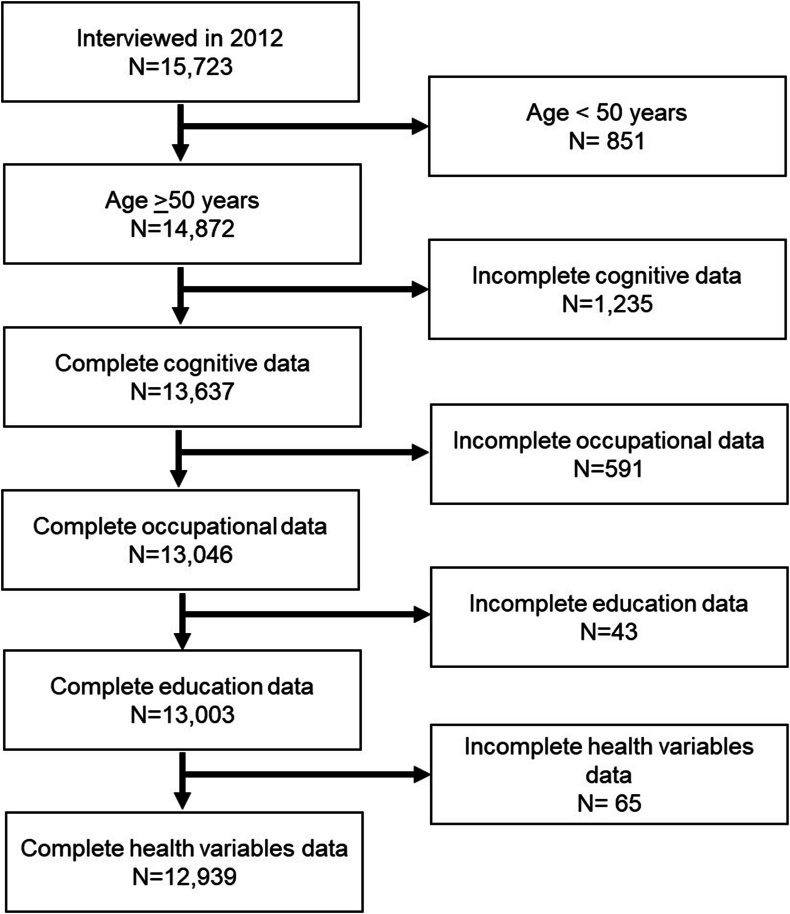
Sample selection of participants interviewed in the 2012 Mexican Health and Aging Study.

We used data from the 2012 MHAS wave because this is the only wave linked to the Occupational Information Network (O∗NET), which is the source for the occupational mental demands data(C.-Y. Li et al., 2023). Fig. 1 illustrates our sample selection process, commencing with a total of 15,723 participants. Our initial step involved selecting individuals aged 50 or older. We then excluded participants with incomplete cognitive test data, even when imputed scores were considered as well as those with non-specific or unreported occupations. Finally, we excluded participants with missing key data on education or health. Our final sample included 12,939 respondents.

## 3 Measures

### 3.1 Occupation

To determine the respondent's main lifetime occupation, we used both closed- and open-ended MHAS questions regarding the details of a respondent's lifetime occupational history. Upon data collection, the MHAS project team analyzes and codes these questions using the Mexican Occupation Classification System, which is formally established and maintained by the Mexican National Institute of Statistics, Geography, and Informatics (INEGI).(Instituto Nacional de Estadística, Geografía e Informática, 2009) The results of this coding linked to the O∗NET provide our occupational variables discussed further below.

### 3.2 Mental demands at work source: O∗NET database

Ideally, we would use data on the mental demands each respondent faced in their main lifetime occupation. Unfortunately, such specific information is not available in the MHAS or any other known sources for Mexico. Consequently, we utilized information from a U.S.-based occupational database known as O∗NET to identify such demands.

O∗NET is based on extensive research into job and organizational analysis, providing detailed specifications regarding the essential attributes and characteristics of both workers and occupations(USDepartment of LaborEmployment and Training Administration, n.d.). These attributes and characteristics, referred to as descriptors, currently encompass data on 923 distinct occupations. Descriptors are organized into two orientations.

1) Worker-Oriented: This dimension revolves around the knowledge, skills, and abilities that individuals need to excel in specific occupations. It offers insights into the competencies required by workers in various roles.
2) Job-Oriented: This dimension delves into how work is performed within these occupations. It provides comprehensive information on tasks, work activities, and other relevant descriptors that characterize the work.

### 3.3 Characterizing occupational mental demands

We used specific descriptors from the O∗NET database to accurately assess the mental demands associated with various occupations. We distinguished between worker and job-oriented mental demands.

For worker-oriented mental demands, we focused on 21 variables categorized under ‘cognitive abilities’ (O∗NET variables 1.A.1.a through 1.A.1.g.2). These variables reflect the skills and capacities expected of workers, particularly their ability to acquire and apply knowledge in problem-solving scenarios.

For job-oriented mental demands, we focused on 10 variables labeled as 'mental processes' (O∗NET variables 4.A.2.a through 4.A.2.b). This category includes work activities that involve processing information, planning, problem-solving, decision-making, and innovating.

We used both these sets of variables because previous research either focused solely on one set, such as job-oriented mental demands(Fisher et al., 2014), or combined variables from both sets(Fujishiro et al., 2019b). To our knowledge, no previous research has considered worker-oriented and job-oriented mental demands as separate descriptors. This separation served as a sensitivity analysis to explore potential differences in how each descriptor associates with cognitive functioning.

Although the O∗NET descriptors and their associated variables were originally developed using US data, their applicability extends beyond the United States. Previous studies have successfully linked these descriptors to data from several other countries, specifically to investigate the impact of occupation on cognitive functioning(Andel et al., 2019; Suemoto et al., 2022; Then et al., 2017; Zülke et al., 2021). These descriptors have also been applied to Mexican data to assess the effects of occupation on survival(Beltrán-Sánchez et al., 2020). For further details on the variables included within each O∗NET descriptor, please refer to Supplementary Table 1.

### 3.4 Creation of mental demands indices

We used variables from the O∗NET database to construct two discrete indices—one focusing on worker-oriented mental demands and the other on job-oriented mental demands. We evaluated the O∗NET descriptors using two scales: importance and level. For our analysis, we used the 'level' scale, which indicates the extent or degree to which a specific skill is necessary for performing an occupation. We calculated each index by computing the mean 'level' scores of the variables within each descriptor. The 'level' scale for each variable is standardized and ranges from 0 to 100.

After calculating these mean scores, we classified them into three demand levels—high, medium, or low—by dividing the score range into three equal parts. Supplementary Table 2 lists specific ranges and cutoff points. This discrete categorization was designed to facilitate classification, analysis, and interpretation of results in combination with education levels. This method aligns with prior research that examined the impact of mental work demands on cognitive function in a German cohort, where O∗NET 'cognitive abilities' variables were averaged and categorized into high, medium, and low levels alongside education categories(Then et al., 2014).

These indices aim to provide a detailed view of cognitive demands across various occupations, aiding in the assessment of their correlation with cognitive health.

### 3.5 Linking MHAS to O∗NET database

We linked MHAS occupations to O∗NET using INEGI occupation code job descriptions. These descriptions were derived from responses to open-ended questions about respondent's activities in their main lifetime occupation, as well as on the place where that occupation was typically performed (e.g., outdoors, or indoors). We achieved this linkage through a modified Delphi technique, which involved the participation of three raters collaborating to establish a unanimous one-to-one correspondence between MHAS and O∗NET occupations. Raters first collectively identified key words from MHAS variables used to search the O∗NET online system and rated the most similar occupation between both data sources. This linkage is currently only available for the 2012 MHAS wave. Detailed information on this linkage process is available in the original project publication(C.-Y. Li et al., 2023).

### 3.6 Education

Building on previous research using the MHAS and aligning with the structural framework of Mexico's educational system, we categorized respondents into three educational levels: (1) ‘low,’ for respondents with no schooling; (2) ‘medium,’ for respondents with 1–6 years of education; and (3) ‘high,’ for respondents with 7 or more years of education(Mejia-Arango et al., 2021).

### 3.7 Cognitive functioning

**Fig. 2:**
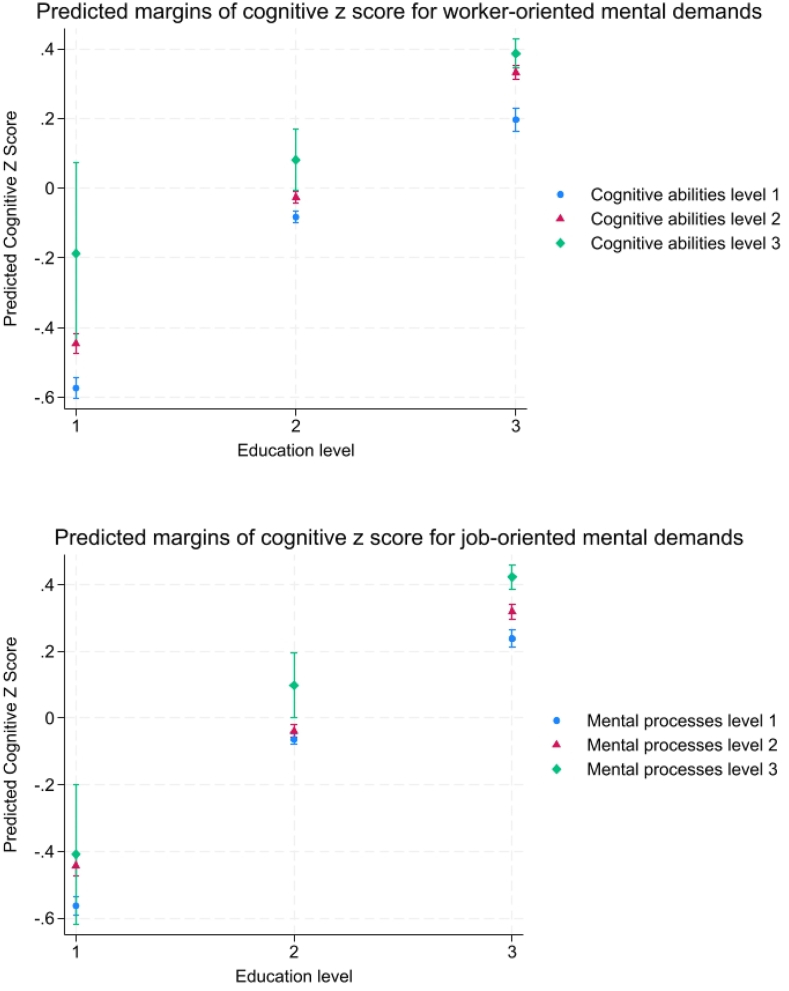
Interaction plot showing the predicted cognitive z-score according to occupational mental demands and education level. Worker-oriented Mental Demands = Cognitive abilities; Job-oriented Mental Demands = Mental processes.

To assess cognitive function, we used tests from the screening portion of the Cross-Cultural Cognitive Examination in the MHAS. These have been shown to be independent of cultural and educational background effects(Glosser et al., 1993). The eight cognitive tests in the MHAS interview are: [1] immediate recall of an 8-word list (0–8 points); [2] delayed recall of an 8-word list (0–8 points); [3] copying a figure (0–2 points); [4] drawing a figure from memory (0–2 points); [5] identifying a target stimulus in a visual array (0–60 points); [6] orientation, ascertained through knowledge of the day, month, and year; [7] semantic verbal fluency, assessed by the naming of animals within a 1-min interval; and [8] numeracy, gauged via a task that involves counting backwards from 20 to 0 within 60 s(Mejía-Arango et al., 2015). We utilized the MHAS datasets that feature imputed scores for cognitive functioning in order to account for missing values. For further details about the imputation procedures please refer to the original publication(Downer et al., 2021). For each cognitive test, we calculate a z-score by comparing the individual's score to the sample mean and standard deviation of all participants who completed a direct interview. We subsequently calculated the mean of the z-scores across all the tests as our measure of overall cognitive functioning(Mejía-Arango et al., 2015).

To ensure our analyses were adjusted properly, we incorporated the demographic and health characteristics noted below.

### 3.8 Demographic characteristics

The MHAS includes variables on respondent age, sex (female/male), community population size (>100,000; 99,000–15,000; 14,999–2500; and <2500), and health insurance (insured/uninsured).

### 3.9 Health characteristics

The MHAS asked participants if they had ever been told by a doctor or medical personnel that they had diabetes or high blood sugar, hypertension or high blood pressure, stroke, or heart attack. We dichotomized each health condition as yes or no. The MHAS assesses depressive symptoms using a modified nine-item version of the Center for Epidemiologic Studies – Depression (CES-D) scale(Radloff, 1977). These items were presented as binary outcomes (yes/no). Following previous research using the MHAS, we classified participants who responded “yes” to five or more items as having depressive symptoms and participants who responded affirmatively to four or fewer items as not presenting depressive symptoms(Saenz et al., 2020).

### 3.10 Statistical analyses

To analyze our data, we used a Generalized Linear Model (GLM) with a Gaussian family and an identity link function(Then et al., 2014). We chose the GLM approach due to its suitability for continuous outcomes like cognitive z-scores, allowing us to estimate changes in cognition in natural units and provide straightforward, interpretable results. This model setup enabled us to examine the association of occupational mental demands (and other covariates) on cognitive functioning, with coefficients offering insights into the expected change in cognitive functioning per unit change in predictors while accounting for all other variables in the model.

#### 3.10.1 Step 1

Initially, we investigated the primary associations of occupational mental demands and educational attainment (both categorized as low, medium, or high) on cognitive functioning. We quantified cognitive functioning using the mean of z-scores from individual cognitive domains, and used age and sex for demographic controls. We conducted three separate analytical models: one using worker-oriented mental demands as the predictor, another using job-oriented mental demands, and a third using educational attainment.

#### 3.10.2 Step 2

In this phase, we expanded our model to include additional demographic and health-related covariates, thereby accounting for the main effects of occupational mental demands and educational attainment. Crucially, we introduced an interaction term to examine the interplay between occupational mental demands and educational levels. For this refined analysis, we computed the predicted mean cognitive z-score margins for each unique combination of occupational mental demands and educational level. We performed this separately for worker-oriented and job-oriented mental demands, allowing us to distinguish their individual impacts on cognitive outcomes. Additionally, to understand the nature of the interaction, we conducted a sequential regression including education with all other controls except occupational mental demands, then conducted another regression introducing occupational mental demands to assess any potential attenuation of education's coefficients. Furthermore, we performed a stratified analysis to explore the relationship between occupational mental demands and cognition within each educational level.

## 4 Results

Table 1 offers a detailed analysis of both sociodemographic and health-related variables, segmented by levels of occupational mental demands. The data demonstrate a significant variance in average cognitive z-scores across these occupational categories. Notably, subgroups with more demanding cognitive occupations register higher average z-scores, indicating higher levels of cognitive demands (Supplementary Fig. 1).

**Table 1:** Sociodemographic and health characteristics. Full sample and by occupational mental demands.

		Occupational mental demands					
	Full sample	Worker-oriented			Job-oriented		
Mean (SD) % column		1	2	3	1	2	3
	12,939	5155 (39.8)	7122 (55)	662 (5.1)	6384 (49.3)	5722 (44.2)	833 (6.4)
Cognitive z-score	−0.019 (0.644)	−0.134 (0.638)	0.025 (0.642)	0.395 (0.451)	−0.083 (0.638)	−0.016 (0.644)	0.456 (0.461)
Education							
No schooling	17.1	21.1	15.6	2	18.8	17.4	2.4
1–6 years	52.4	62.8	48.2	17.7	60.7	49.3	11
7 or more	30.4	16	36.2	80.4	20.5	33.3	86.6
Age	64.8 (9.4)	64.4 (9.3)	65.1 (9.5)	63.4 (8.5)	64.3 (9.3)	65.6 (9.5)	62.9 (8.4)
Sex Female	56.9	80.5	41.6	38.7	78.5	34.1	48.1
Locality Size							
100,000	59	59.7	56.5	80.5	61.5	53.1	79.7
15,000–99,999	11	11.3	10.9	10.4	11.2	11	10.6
2500 - 14,999	10.8	10.6	11.4	5.44	10.4	12.15	4.3
<2500	19.2	18.4	21.2	3.6	16.9	23.8	5.4
Hypertension	44.2	48.6	41.3	40	48.2	40.2	40.8
Diabetes	23.2	26.7	20.8	21.3	26.5	20	19.9
Heart attack	3.6	3.1	3.8	4.8	3.2	3.8	4.6
Stroke	2	1.9	2.1	1.7	1.9	2.2	0.8
Depression	44.6	50	42.1	30.2	49.2	41.6	30.3
Health insurance	87.8	88	87.1	93.5	88.2	86.4	94.7

We find that groups facing greater occupational mental demands are more likely to have individuals with higher educational attainment. The median age is similar across different occupational categories while women are more likely to be in less cognitively demanding occupations. Additionally, those working in occupations requiring higher mental demands are more concentrated in urban settings (Table 1).

We also find that persons with higher rates of hypertension and diabetes are more prevalent in less mentally demanding occupations. The data also indicate a higher frequency of heart attacks among those with more mentally taxing jobs, while stroke incidence was similar across all occupational categories. There were lower levels of depression among those in more mentally demanding roles, while health insurance coverage was most prominent among those with greater occupational cognitive requirements.

In the initial phase of our analysis, we used Generalized Linear Models to examine the impact of occupational mental demands and educational attainment on cognitive functioning (Table 2). We found a strong positive correlation between elevated cognitive z-scores and high worker-oriented (β = 0.50; CI = 0.45, 0.55) and job-oriented (β = 0.49; CI = 0.45, 0.53) mental demands relative to low ones. The influence of high educational attainment on cognitive z-scores was even more pronounced (β = 0.90; CI = 0.87, 0.92). In other words, this analysis showed those with high worker-oriented mental demands, high job-oriented mental demands, or high levels of educational attainment had better cognitive performance than others.

**Table 2:** Generalized linear model analysis on the association of occupational mental demands and educational attainment on cognitive functioning.

	Worker-oriented Mental Demands (Cognitive abilities)		Job-oriented Mental Demands (Mental processes)		Education	
	Coefficient	95% CI	Coefficient	95% CI	Coefficient	95% CI
Level						
Low	Reference		Reference		Reference	
Medium	0.186	0.166, 0.208	0.099	0.078, 0.121	0.486	0.462, 0.509
High	0.500	0.455, 0.546	0.487	0.446, 0.527	0.896	0.87, 0.923

Age	−0.033	−0.034, −0.032	−0.033	−0.034, −0.032	−0.024	−0.024, −0.023
Sex (ref: male)	0.010	−0.011, 0.031	−0.021	−0.042, 0.000	−0.023	−0.04, −0.006

In the second phase of our analysis, we examined the relationships between cognitive functioning and an array of sociodemographic and health-related variables (Table 3). We operationalized two models, which varied only in the measurement of occupational mental demands. Model 1 employed worker-oriented mental demands and Model 2 used job-oriented mental demands. In both models, educational attainment remained a potent predictor, revealing a significant positive association with higher cognitive z-scores (Model 1: β = 0.49, CI = 0.46, 0.52 for medium-level education; β = 0.77, CI = 0.73, 0.81 for high-level education. Model 2: β = 0.5, CI = 0.47, 0.53 for medium-level education; β = 0.80, CI = 0.76, 0.84 for high-level education).

**Table 3:** Generalized linear model analysis on the association of occupational mental demands and educational attainment on cognitive functioning. Models include interaction terms between occupational mental demands and education levels, and additional demographic and health covariates.

Model 1: Worker-oriented Mental Demands (Cognitive abilities)		Model 2: Job-oriented Mental Demands (Mental processes)	
	Coefficient [95% CI]		Coefficient [95% CI]
Medium cognitive abilities	0.13 [0.09, 0.17]	Medium mental processes	0.12 [0.08, 0.16]
High cognitive abilities	0.39 [0.12, 0.65]	High mental processes	0.15 [-0.06, 0.37]
Medium education	0.49 [0.46, 0.52]	Medium education	0.50 [0.47, 0.53]
High education	0.77 [0.73, 0.82]	High education	0.80 [0.76, 0.84]
Medium cognitive abilities x medium education	−0.07 [-0.12, −0.02]	Medium mental processes x medium education	−0.10 [-0.14, −0.05]
Medium cognitive abilities x high education	0.01 [-0.05, 0.06]	Medium mental processes x high education	−0.04 [-0.09, 0.01]
High cognitive abilities x medium education	−0.22 [-0.50, 0.05]	High mental processes x medium education	0.01 [-0.23, 0.24]
High cognitive abilities x high education	−0.20 [-0.46, 0.07]	High mental processes x high education	0.03 [-0.19, 0.25]
Age	−0.02 [-0.03, −0.02]	Age	−0.02 [-0.03, −0.02]
Female	0.01 [-0.01, 0.03]	Female	0.00 [-0.02, 0.02]
Pop 15,000–99,999	−0.03 [-0.05, 0.00]	Pop 15,000–99,999	−0.03 [-0.05, 0.00]
Pop 2500 - 14,999	−0.09 [-0.11, −0.06]	Pop 2500 - 14,999	−0.08 [-0.11, −0.06]
Pop <2500	−0.16 [-0.18, −0.13]	Pop <2500	−0.16 [-0.18, −0.13]
No hypertension	0.02 [0.00, −0.03]	No hypertension	0.02 [-0.00, 0.03]
No diabetes	−0.03 [-0.05, −0.01]	No diabetes	−0.03 [-0.05, 0.03]
No heart attack	0.04 [-0.01, 0.08]	No heart attack	0.04 [-0.01, 0.08]
No stroke	−0.17 [-0.23, −0.11]	No stroke	−0.16 [-0.22, −0.10]
No depression	−0.07 [-0.08, −0.05]	No depression	−0.06 [-0.08, −0.05]
No health insurance	0.05 [0.02, 0.08]	No health insurance	0.05 [0.02, 0.07]

In Model 1, which uses worker-oriented mental demands, higher levels of mental demands were associated with stronger positive effects on cognitive z-scores. Specifically, relative to individuals with low educational attainment, medium worker-oriented mental demands were associated with a 0.13-unit increase in cognitive z-scores (β = 0.13; 95% CI = 0.09, 0.17), and high worker-oriented mental demands were associated with an even larger increase of 0.39 units (β = 0.39; 95% CI = 0.12, 0.65). In Model 2, which examines job-oriented mental demands, there was a significant positive association only medium mental processes and cognitive z-scores (β = 0.12; 95% CI = 0.08, 0.16) relative to low mental demands.

Both models showed a statistically significant negative interaction between medium mental demands and medium education level. The interaction effect was more pronounced in the job-oriented mental demands model (β = −0.1; 95% CI = −0.14, −0.05) than in the worker-oriented model (β = −0.07; 95% CI = −0.12, −0.02). The remaining interaction terms in both models were inconsistent and statistically insignificant.

Both models consistently indicated that age had a negative association with cognitive functioning (β = −0.02; CI = −0.03, −0.02). Residing in smaller localities (population <2500) also negatively impacted cognitive z-scores (β = −0.16; CI = −0.18, −0.13). The health-related variables generally showed mixed results in both models, but having a previous history of stroke was associated with a lower cognitive z-score in both cases (Model 1: β = −0.17, CI = −0.23, −0.11; Model 2: β = −0.16, CI = −0.23, −0.10). Having depressive symptoms was associated with lower cognitive functioning (Model 1: β = −0.07, CI = −0.08, −0.05; Model 2: β = −0.06, CI = −0.08, −0.05), while being insured exhibited a positive association (Model 1: β = 0.05, CI = 0.02, 0.07; Model 2: β = 0.05, CI = 0.02, 0.07) (Table 3).

The interaction between occupational mental demands and education level shows complex associations with cognitive functioning. High education level predicted a positive cognitive z-score regardless of occupational mental demands (Fig. 2). This includes participants with low occupational mental demands and high education who have higher predicted z-scores (worker-oriented = 0.2, CI = 0.16, 0.23; job-oriented = 0.24, CI = 0.21, 0.26) than participants with high occupational mental demands and medium education (worker-oriented = 0.08, CI = −0.0, 0.16; job-oriented = 0.1, CI = −0.0, 0.2). Low education levels predicted negative cognitive z-scores even for those with high mental demands (worker-oriented = −0.19, CI = −0.45, 0.07; job-oriented = −0.41, CI = −0.62, −0.2).

Higher occupational mental demands were associated with higher cognitive z-scores at each educational level. In the case of worker-oriented mental demands, moving from low to medium demands resulted in significantly higher cognitive z-scores at all education levels (Fig. 2).

In the sequential regression, we did not observe any significant attenuation between education and higher cognitive z-scores when introducing occupational mental demands (Supplementary Table 3). Occupational mental demands, however, were significantly associated with higher cognitive z-scores.

In the model exploring the relationship between occupational mental demands and cognition stratified by education level (Supplementary Table 4), higher mental demands were significantly associated with higher cognitive z-scores. For those with high education, medium worker-oriented mental demands were associated with a 0.14 (CI = 0.11, 0.17) increase in cognitive z-score, and high mental demands were associated with a 0.20 (CI = 0.16, 0.25) increase. In the low education stratum, only high worker-oriented mental demands were significantly associated with a higher cognitive z-score (β = 0.34; CI = 0.01, 0.67). Other mental demands in the low education level stratum were not significantly associated with higher cognitive z-scores.

## 5 Discussion

The current study examines the relationship between educational attainment, occupational mental demands, and cognitive functioning, controlling for sociodemographic and health-related variables. It contributes to a growing body of evidence noting the primacy of both education and mental occupational demands in shaping cognitive outcomes.

Participants with higher levels of educational attainment consistently demonstrated superior cognitive performance. Occupations that required the highest educational qualifications included professionals; educators; directors in the public, private, and social sectors; administrative support staff; and department heads, coordinators, and supervisors in administrative and service activities (Supplementary Fig. 2). Previous research has found that education may increase regional cortical thickness in areas such as the transverse temporal cortex, insula, and isthmus of the cingulate cortex, thereby enhancing brain reserve and contributing to better cognitive performance among more educated individuals(Liu et al., 2012). Other research links educational attainment to improved cognitive function in later life stages, suggesting that education plays a critical role in maintaining cognitive health as people age(Clouston et al., 2020).

The data also show a strong positive correlation between cognitive functioning and occupational mental demands, measured through both job-oriented mental processes and worker-oriented cognitive abilities. Occupations requiring higher levels of cognitive engagement yield individuals with better cognitive z-scores. This underscores the cognitive advantages that accrue over a lifetime of engaging in mentally intricate tasks, and it adds an additional layer of evidence to the potential benefits of mentally stimulating work environments(Lee et al., 2022). Studies have shown that higher cognitive stimulation at work is associated with lower levels of proteins that inhibit central nervous system axonogenesis and synaptogenesis, thereby reducing the risk of dementia(Kivimäki et al., 2021). This suggests that cognitively stimulating work environments may help maintain neural networks and prevent cognitive impairment. INEGI job categories with the highest mental demands were Department Heads, Coordinators, and Supervisors in Administrative and Service Activities; Technicians; Educators; and Professionals (Supplementary Fig. 2).

In addition, we found no meaningful difference between worker-oriented and job-oriented mental demands when assessing the impact of occupational mental demands on cognition. While worker-oriented mental demands displayed a slightly stronger association with elevated cognitive z-scores, they were more challenging to measure requiring 21 variables compared to the 10 needed to assess job-oriented mental demands (Supplementary Table 1). While previous research has utilized various O∗NET descriptors to represent occupational mental demands,(Then et al., 2017), our study reaffirms the efficacy of the variables encompassed within the worker-oriented and job-oriented mental demands descriptors. This finding is in line with previous studies, confirming the mental demands descriptors as robust measures of occupational mental demands(Fisher et al., 2014).

Perhaps most intriguingly, we identified a statistically significant negative interaction between medium-level educational attainment and medium-level occupational mental demands. We found 84% of respondents in medium-level levels of education and mental demands were employed in Agriculture, Livestock, Forestry, and Fishing; Artisans and Workers in Production, Repair, and Maintenance; or Merchants and Sales Representatives sectors (Supplementary Fig. 2). One possible explanation is that sustained cognitive engagement is crucial for maintaining cognitive functioning over time(Bielak & Gow, 2023). When the mental activities from educational or occupational environments are not continued, the benefits in cognitive capacities may be attenuated or even lost(Hyun et al., 2022). This relation may be influenced by the type of occupation, especially if the associated activities are not cognitively challenging or innovative. Thus, the lack of ongoing cognitive stimulation in certain occupations could lead to diminished cognitive benefits over time.

Interactions for other combinations of education and occupational demands were not statistically significant. For other occupations, the association of mental demands on cognition appeared to be largely independent of education, a finding that matches previous research(Then et al., 2014). This independence can be understood from a life course perspective: education influences cognition during and after early developmental periods, whereas occupational demands typically begin affecting cognition in young adulthood and continue until retirement. Both educational and occupational attainment contribute to cognitive reserve, but they may engage different underlying brain processes(Stern et al., 2019).

Engaging in cognitively stimulating activities later in life can provide compensatory cognitive benefits for individuals with low educational attainment(Lachman et al., 2010). Thus, cognitive reserve is not a static condition; it can be influenced and enhanced at any point in a person's lifetime. However, the influence of educational attainment on cognitive outcomes is demonstrably stronger. Consequently, individuals with higher levels of educational attainment are better positioned to achieve superior cognitive outcomes than those who rely solely on occupations with high mental demands.

The sequential and stratified analyses support a life course approach, demonstrating that the relationship between education and cognition remains largely unaffected by occupation, while the association between occupation and better cognitive outcomes remains significant.

Another relevant finding was the higher rates of hypertension and diabetes observed in groups with less mentally demanding occupations. Previous research has shown that high-skilled occupations tend to have a better risk profile compared to low-skilled occupations. Specifically, those in high-skilled occupations have lower rates of obesity, type 2 diabetes, and hypertension. This difference can be attributed to higher socioeconomic status and healthier behaviors, including lower smoking rates (Väisänen et al., 2020). We also observed decreased levels of depression among those in more mentally demanding roles. This supports previous evidence of a positive association between higher occupational prestige and lower depression levels. These mental health benefits are directly influenced by a supportive work environment and lower work-related stress(Heinz et al., 2018), and indirectly by higher socioeconomic status(Fujishiro et al., 2010).

While this study provides valuable insights on educational attainment, occupational mental demands, and cognitive functioning, it has several limitations. First, some MHAS respondents lack data on cognition and employment, potentially introducing selection bias into our analysis. Second, we cannot exclude the possibility of reverse causality; individuals with superior cognitive function may be more likely to pursue occupations with higher mental demands, thereby complicating the interpretation of our results. Third, our research relies on cross-sectional data as the match between MHAS and O∗NET occupations is only available for the 2012 wave. This prevents us from examining the longitudinal effects of occupational characteristics on cognitive decline. In particular, there may be unobserved transient characteristics that influence cognitive performance at the single point in time we assessed. Fourth, the MHAS includes a single question about each respondent's 'main' job, rather than a comprehensive occupational history. Given that individuals often engage in multiple jobs throughout their careers, the cognitive impacts of their work may vary by the time they spend in different positions and the sequence of different jobs held. A more detailed analysis of occupational histories, employing a larger sample with longitudinal data and finer categorization of occupations, would allow future researchers to better assess the influence of various work trajectories on cognitive functioning in older adults.

Despite its limitations, our work provides a robust and nuanced understanding of the interplay between educational attainment, occupational cognitive demands, and cognitive functioning across a wide range of sociodemographic and health-related variables. Our findings substantiate the critical role of education in cognitive resilience, as well as the contributions of mentally demanding occupations in enhancing cognitive performance. While our analysis reaffirms the independent relation of both educational attainment and occupational mental demands with cognitive outcomes, it highlights that the influence of education on cognition is markedly stronger. Our results expand the existing literature by providing a multidimensional view of life course factors associated with cognitive health in a middle-income country.

Future longitudinal research is necessary to better delineate causal relationships between cognition and social context. We also suggest further exploration of the underlying mechanisms that may explain the observed effects of lifelong exposure to cognition stimulating activities. Although limited by certain methodological constraints, our findings offer important implications for public policy and interventions aimed at fostering cognitive well-being across diverse populations.

## CRediT authorship contribution statement

**José Eduardo Cabrero Castro:** Writing – review & editing, Writing – original draft, Visualization, Validation, Supervision, Methodology, Investigation, Formal analysis, Data curation, Conceptualization. **Mariela Gutierrez:** Writing – review & editing, Writing – original draft, Visualization, Validation, Methodology, Investigation, Data curation, Conceptualization. **Theresa Andrasfay:** Writing – review & editing, Writing – original draft, Visualization, Validation, Methodology, Investigation, Conceptualization. **Emma Aguila:** Writing – review & editing, Writing – original draft, Visualization, Validation, Methodology, Investigation, Conceptualization. **Brian Downer:** Writing – review & editing, Writing – original draft, Visualization, Validation, Supervision, Methodology, Funding acquisition, Conceptualization.

## Ethical statement

This research was a secondary analysis of de-identified data previously collected by the MHAS and is publicly available. Ethical approval and consent to participate are not required for this study.

The University of Texas Medical Branch Institutional Review Board and the National Institute of Statistics and Geography, and the National Institute of Public Health in Mexico have approved the MHAS study procedures and survey instruments.

## Declaration of competing interest

The authors declare that they have no known competing financial interests or personal relationships that could have appeared to influence the work reported in this paper.

## Data Availability

The data utilized in this study is sourced from the Mexican Health and Aging Study. It is publicly accessible upon registration on the MHAS official website: https://mhasweb.org/Home/index.aspx.

## References

[bib1] Andel R., Dávila-Roman A.L., Grotz C., Small B.J., Markides K.S., Crowe M. (2019). Complexity of work and incident cognitive impairment in Puerto Rican older adults. Journals of Gerontology Series B: Psychological Sciences and Social Sciences.

[bib2] Baldivia B., Andrade V.M., Bueno O.F.A. (2008). Contribution of education, occupation and cognitively stimulating activities to the formation of cognitive reserve. Dement Neuropsychol.

[bib3] Beltrán-Sánchez H., Goldman N., Pebley A.R., Morales J.F. (2020). Calloused hands, shorter life? Occupation and older-age survival in Mexico. Demographic Research.

[bib4] Ben-Shlomo Y., Kuh D. (2002). A life course approach to chronic disease epidemiology: Conceptual models, empirical challenges and interdisciplinary perspectives. International Journal of Epidemiology.

[bib5] Bielak A.A.M., Gow A.J. (2023). A decade later on how to “use it” so we don't “lose it”: An update on the unanswered questions about the influence of activity participation on cognitive performance in older age. Gerontology.

[bib6] Cabrero-Castro J.E., Mehta N., Wong R., Downer B. (2023). Cognitive life expectancy by educational attainment in Mexican adults aged 60 and older. Salud Pública de México.

[bib7] Chapko D., McCormack R., Black C., Staff R., Murray A. (2018). Life-course determinants of cognitive reserve (CR) in cognitive aging and dementia – a systematic literature review. Aging & Mental Health.

[bib8] Clouston S.A.P., Smith D.M., Mukherjee S., Zhang Y., Hou W., Link B.G., Richards M. (2020). Education and cognitive decline: An integrative analysis of global longitudinal studies of cognitive aging. Journals of Gerontology Series B: Psychological Sciences and Social Sciences.

[bib9] CONAPO (2004). Envejecimiento de la población de México: Reto del Siglo XXI | Consejo Nacional de Población CONAPO. http://www.conapo.gob.mx/es/CONAPO/Envejecimiento_de_la_poblacion_de_Mexico__reto_del_Siglo_XXI.

[bib10] Creighton M., Park H. (2010). Closing the gender gap: Six decades of reform in Mexican education. Comparative Education Review.

[bib11] Cruz C.A.F., Álvarez J.L., Aguirre J.F.I., Sánchez F.J.Z., Ovalle R.I.A., Martínez W.C., Campos M.Á.S. (2015). Análisis de los microdatos del censo de 1930: A 80 años del México posrevolucionario. INTERNATIONAL JOURNAL OF STATISTICS AND GEOGRAPHY.

[bib12] Díaz-Venegas C., Samper-Ternent R., Michaels-Obregón A., Wong R. (2019). The effect of educational attainment on cognition of older adults: Results from the Mexican Health and Aging Study 2001 and 2012. Aging & Mental Health.

[bib13] Downer B., Avila J., Chen N.-W., Wong R. (2021). Imputation procedures for cognitive variables in the Mexican health and aging study: Evaluating the bias from excluding participants with missing data. Realidad, Datos Y Espacio: Revista Internacional De Estadistica Y Geografia.

[bib14] Downer B., Gutierrez M., Arango S.M., Wong R. (2022). Cohort differences in early-life socioeconomic status and late-life cognitive impairment in Mexico. Innovation in Aging.

[bib15] Finkel D., Andel R., Gatz M., Pedersen N.L. (2009). The role of occupational complexity in trajectories of cognitive aging before and after retirement. Psychology and Aging.

[bib16] Fisher G.G., Stachowski A., Infurna F.J., Faul J.D., Grosch J., Tetrick L.E. (2014). Mental work demands, retirement, and longitudinal trajectories of cognitive functioning. Journal of Occupational Health Psychology.

[bib17] Fujishiro K., MacDonald L.A., Crowe M., McClure L.A., Howard V.J., Wadley V.G. (2019). The role of occupation in explaining cognitive functioning in later life: Education and occupational complexity in a U.S. National sample of black and white men and women. Journals of Gerontology Series B: Psychological Sciences and Social Sciences.

[bib18] Fujishiro K., MacDonald L.A., Crowe M., McClure L.A., Howard V.J., Wadley V.G. (2019). The role of occupation in explaining cognitive functioning in later life: Education and occupational complexity in a U.S. National sample of black and white men and women. Journals of Gerontology Series B: Psychological Sciences and Social Sciences.

[bib19] Fujishiro K., Xu J., Gong F. (2010). What does “occupation” represent as an indicator of socioeconomic status?: Exploring occupational prestige and health. Social Science & Medicine.

[bib20] García B., Oliveira O. de, García B., Oliveira O. de (2001). Cambios socioeconómicos y división del trabajo en las familias mexicanas. Investigación Económica.

[bib21] Glosser G., Wolfe N., Albert M.L., Lavine L., Steele J.C., Calne D.B., Schoenberg B.S. (1993). Cross-cultural cognitive examination: Validation of a dementia screening instrument for neuroepidemiological research. Journal of the American Geriatrics Society.

[bib22] Gonçalves N.G., Avila J.C., Bertola L., Obregón A.M., Ferri C.P., Wong R., Suemoto C.K. (2023). Education and cognitive function among older adults in Brazil and Mexico. Alzheimer's and Dementia.

[bib23] Gutiérrez P.S. (2012).

[bib24] Heinz A.J., Meffert B.N., Halvorson M.A., Blonigen D., Timko C., Cronkite R. (2018). Employment characteristics, work environment, and the course of depression over 23 years: Does employment help foster resilience?. Depression and Anxiety.

[bib25] Hyun J., Hall C.B., Katz M.J., Derby C.A., Lipnicki D.M., Crawford J.D., Guaita A., Vaccaro R., Davin A., Kim K.W., Han J.W., Bae J.B., Röhr S., Riedel-Heller S., Ganguli M., Jacobsen E., Hughes T.F., Brodaty H., Kochan N.A., Lipton R.B. (2022). Education, occupational complexity, and incident dementia: A cosmic collaborative cohort study. Journal of Alzheimer's Disease.

[bib26] Instituto Nacional de Estadística, Geografía e Informática (2009). https://www.inegi.org.mx/contenidos/clasificadoresycatalogos/doc/clasificacion_mexicana_de_ocupaciones_vol_i.pdf.

[bib27] Kivimäki M., Walker K.A., Pentti J., Nyberg S.T., Mars N., Vahtera J., Suominen S.B., Lallukka T., Rahkonen O., Pietiläinen O., Koskinen A., Väänänen A., Kalsi J.K., Goldberg M., Zins M., Alfredsson L., Westerholm P.J.M., Knutsson A., Theorell T., Lindbohm J.V. (2021). Cognitive stimulation in the workplace, plasma proteins, and risk of dementia: Three analyses of population cohort studies. BMJ.

[bib28] Kuh D. (2003). Life course epidemiology. Journal of Epidemiology & Community Health.

[bib29] Lachman M.E., Agrigoroaei S., Murphy C., Tun P.A. (2010). Frequent cognitive activity compensates for education differences in episodic memory. American Journal of Geriatric Psychiatry.

[bib30] Lee Y.J., Gonzales E., Andel R. (2022). Multifaceted demands of work and cognitive functioning: Findings from the health and retirement study. Journals of Gerontology Series B: Psychological Sciences and Social Sciences.

[bib31] Li C.-Y., Aguila E., Arthur P., Peniche J., Gútierrez M., Hernández M., Wong R. (2023). Assigning lifetime occupation domains for older Mexicans: MHAS-O∗NET linkage protocol. Salud Pública de México.

[bib32] Li X., Song R., Qi X., Xu H., Yang W., Kivipelto M., Bennett D.A., Xu W. (2021). Influence of cognitive reserve on cognitive trajectories: Role of brain pathologies. Neurology.

[bib33] Liu Y., Julkunen V., Paajanen T., Westman E., Wahlund L.-O., Aitken A., Sobow T., Mecocci P., Tsolaki M., Vellas B., Muehlboeck S., Spenger C., Lovestone S., Simmons A., Soininen H., AddNeuroMed C. (2012). Education increases reserve against Alzheimer's disease—evidence from structural MRI analysis. Neuroradiology.

[bib34] Medellín R.A., Izquierdo C.M. (2012). Incremento de la población, capacitación y empleo en México (1960-1970). Revista Latinoamericana de Estudios Educativos.

[bib35] Mejia-Arango S., Avila J., Downer B., Garcia M.A., Michaels-Obregon A., Saenz J.L., Samper-Ternent R., Wong R. (2021). Effect of demographic and health dynamics on cognitive status in Mexico between 2001 and 2015: Evidence from the Mexican health and aging study. Geriatrics.

[bib36] Mejía-Arango S., Wong R., Michaels-Obregón A. (2015). Normative and standardized data for cognitive measures in the Mexican Health and Aging Study. Salud Publica De Mexico.

[bib37] Pearlman S., Rubb S. (2020). The impact of education-occupation mismatches on wages in Mexico. Applied Economics Letters.

[bib38] Prince M., Acosta D., Albanese E., Arizaga R., Ferri C.P., Guerra M., Huang Y., Jacob K.S., Jimenez-Velazquez I.Z., Rodriguez J.L., Salas A., Sosa A.L., Sousa R., Uwakwe R., van der Poel R., Williams J., Wortmann M. (2008). Ageing and dementia in low and middle income countries-Using research to engage with public and policy makers. International Review of Psychiatry.

[bib39] Prince M., Acosta D., Ferri C.P., Guerra M., Huang Y., Llibre Rodriguez J.J., Salas A., Sosa A.L., Williams J.D., Dewey M.E., Acosta I., Jotheeswaran A.T., Liu Z. (2012). Dementia incidence and mortality in middle-income countries, and associations with indicators of cognitive reserve: A 10/66 dementia research group population-based cohort study. Lancet (London, England).

[bib40] Prince M., Bryce R., Albanese E., Wimo A., Ribeiro W., Ferri C.P. (2013). The global prevalence of dementia: A systematic review and metaanalysis. Alzheimer's and Dementia.

[bib41] Prince M.J., de Rodriguez J.L., Noriega L., Lopez A., Acosta D., Albanese E., Arizaga R., Copeland J.R.M., Dewey M., Ferri C.P., Guerra M., Huang Y., Jacob K.S., Krishnamoorthy E.S., McKeigue P., Sousa R., Stewart R.J., Salas A., Sosa A.L., 10/66 Dementia research group (2008). The 10/66 dementia research group's fully operationalised DSM-IV dementia computerized diagnostic algorithm, compared with the 10/66 dementia algorithm and a clinician diagnosis: A population validation study. BMC Public Health.

[bib42] Radloff L.S. (1977). The CES-D scale: A self-report depression scale for research in the general population. Applied Psychological Measurement.

[bib43] Saenz J.L., Garcia M.A., Downer B. (2020). Late life depressive symptoms and cognitive function among older Mexican adults: The past and the present. Aging & Mental Health.

[bib44] Smart E.L. (2015). Occupational complexity and lifetime cognitive abilities. Neurology.

[bib45] Stern Y., Barnes C.A., Grady C., Jones R.N., Raz N. (2019). Brain reserve, cognitive reserve, compensation, and maintenance: Operationalization, validity, and mechanisms of cognitive resilience. Neurobiology of Aging.

[bib46] Suemoto C.K., Bertola L., Grinberg L.T., Leite R.E.P., Rodriguez R.D., Santana P.H., Pasqualucci C.A., Jacob-Filho W., Nitrini R. (2022). Education, but not occupation, is associated with cognitive impairment: The role of cognitive reserve in a sample from a low-to-middle-income country. Alzheimer's and Dementia: The Journal of the Alzheimer’s Association.

[bib47] Then F.S., Luck T., Heser K., Ernst A., Posselt T., Wiese B., Mamone S., Brettschneider C., König H.-H., Weyerer S., Werle J., Mösch E., Bickel H., Fuchs A., Pentzek M., Maier W., Scherer M., Wagner M., Riedel-Heller S.G., AgeCoDe Study Group (2017). Which types of mental work demands may be associated with reduced risk of dementia?. Alzheimer's and Dementia: The Journal of the Alzheimer’s Association.

[bib48] Then F.S., Luck T., Luppa M., Arélin K., Schroeter M.L., Engel C., Löffler M., Thiery J., Villringer A., Riedel-Heller S.G. (2014). Association between mental demands at work and cognitive functioning in the general population – results of the health study of the Leipzig research center for civilization diseases (LIFE). Journal of Occupational Medicine and Toxicology.

[bib49] U.S. Department of Labor, Employment and Training Administration (USDOL/ETA). (n.d.). The O∗NET® Content Model. O∗NET Resource Center. Retrieved September 11, 2023, from www.onetcenter.org/content.html.

[bib50] Väisänen D., Kallings L.V., Andersson G., Wallin P., Hemmingsson E., Ekblom-Bak E. (2020). Lifestyle-associated health risk indicators across a wide range of occupational groups: A cross-sectional analysis in 72,855 workers. BMC Public Health.

[bib51] Wong R., Michaels-Obregon A., Palloni A. (2017). Cohort profile: The Mexican health and aging study (MHAS). International Journal of Epidemiology.

[bib52] Wong R., Palloni A., Uhlenberg P. (2009). International handbook of population aging.

[bib53] Zeki Al Hazzouri A., Haan M.N., Kalbfleisch J.D., Galea S., Lisabeth L.D., Aiello A.E. (2011). Life-course socioeconomic position and incidence of dementia and cognitive impairment without dementia in older Mexican Americans: Results from the sacramento area latino study on aging. American Journal of Epidemiology.

[bib54] Zülke A.E., Luppa M., Röhr S., Weißenborn M., Bauer A., Samos F.-A.Z., Kühne F., Zöllinger I., Döhring J., Brettschneider C., Oey A., Czock D., Frese T., Gensichen J., Haefeli W.E., Hoffmann W., Kaduszkiewicz H., König H.-H., Thyrian J.R., Riedel-Heller S.G. (2021). Association of mental demands in the workplace with cognitive function in older adults at increased risk for dementia. BMC Geriatrics.

