# Supplementary material for: The dual impact of education and occupation on cognitive functioning in older Mexican adults: A cross-sectional exploratory study
Source: SSM Popul Health. 2024 Dec 13;29:101738. doi: 10.1016/j.ssmph.2024.101738 (PMC11721833; doi:10.1016/j.ssmph.2024.101738)
Supplement: Multimedia component 1 [file mmc1.docx]

Supplementary table 1. List of variables contained in the worker-oriented (cognitive abilities) and job-oriented (mental processes) O*NET descriptors. Bold shows the main descriptor.

| O*NET ID | Variable | Description |
| --- | --- | --- |
|  |  |  |
| **1.A.1** | **Worker-oriented mental demands Cognitive Abilities** | **Abilities that influence the acquisition and application of knowledge in problem solving** |
| 1.A.1.a | Verbal Abilities | Abilities that influence the acquisition and application of verbal information in problem solving |
| 1.A.1.a.1 | Oral Comprehension | The ability to listen to and understand information and ideas presented through spoken words and sentences. |
| 1.A.1.a.2 | Written Comprehension | The ability to read and understand information and ideas presented in writing. |
| 1.A.1.a.3 | Oral Expression | The ability to communicate information and ideas in speaking so others will understand. |
| 1.A.1.a.4 | Written Expression | The ability to communicate information and ideas in writing so others will understand. |
| 1.A.1.b | Idea Generation and Reasoning Abilities | Abilities that influence the application and manipulation of information in problem solving |
| 1.A.1.b.1 | Fluency of Ideas | The ability to come up with a number of ideas about a topic (the number of ideas is important, not their quality, correctness, or creativity). |
| 1.A.1.b.2 | Originality | The ability to come up with unusual or clever ideas about a given topic or situation, or to develop creative ways to solve a problem. |
| 1.A.1.b.3 | Problem Sensitivity | The ability to tell when something is wrong or is likely to go wrong. It does not involve solving the problem, only recognizing that there is a problem. |
| 1.A.1.b.4 | Deductive Reasoning | The ability to apply general rules to specific problems to produce answers that make sense. |
| 1.A.1.b.5 | Inductive Reasoning | The ability to combine pieces of information to form general rules or conclusions (includes finding a relationship among seemingly unrelated events). |
| 1.A.1.b.6 | Information Ordering | The ability to arrange things or actions in a certain order or pattern according to a specific rule or set of rules (e.g., patterns of numbers, letters, words, pictures, mathematical operations). |
| 1.A.1.b.7 | Category Flexibility | The ability to generate or use different sets of rules for combining or grouping things in different ways. |
| 1.A.1.c | Quantitative Abilities | Abilities that influence the solution of problems involving mathematical relationships |
| 1.A.1.c.1 | Mathematical Reasoning | The ability to choose the right mathematical methods or formulas to solve a problem. |
| 1.A.1.c.2 | Number Facility | The ability to add, subtract, multiply, or divide quickly and correctly. |
| 1.A.1.d | Memory | Abilities related to the recall of available information |
| 1.A.1.d.1 | Memorization | The ability to remember information such as words, numbers, pictures, and procedures. |
| 1.A.1.e | Perceptual Abilities | Abilities related to the acquisition and organization of visual information |
| 1.A.1.e.1 | Speed of Closure | The ability to quickly make sense of, combine, and organize information into meaningful patterns. |
| 1.A.1.e.2 | Flexibility of Closure | The ability to identify or detect a known pattern (a figure, object, word, or sound) that is hidden in other distracting material. |
| 1.A.1.e.3 | Perceptual Speed | The ability to quickly and accurately compare similarities and differences among sets of letters, numbers, objects, pictures, or patterns. The things to be compared may be presented at the same time or one after the other. This ability also includes comparing a presented object with a remembered object. |
| 1.A.1.f | Spatial Abilities | Abilities related to the manipulation and organization of spatial information |
| 1.A.1.f.1 | Spatial Orientation | The ability to know your location in relation to the environment or to know where other objects are in relation to you. |
| 1.A.1.f.2 | Visualization | The ability to imagine how something will look after it is moved around or when its parts are moved or rearranged. |
| 1.A.1.g | Attentiveness | Abilities related to application of attention |
| 1.A.1.g.1 | Selective Attention | The ability to concentrate on a task over a period of time without being distracted. |
| 1.A.1.g.2 | Time Sharing | The ability to shift back and forth between two or more activities or sources of information (such as speech, sounds, touch, or other sources). |
|  | | |
| **4.A.2** | **Job-oriented mental demands**  **Mental Processes** | **What processing, planning, problem-solving, decision-making, and innovating activities are performed with job-relevant information?** |
| 4.A.2.a | Information and Data Processing | How is information processed to perform this job? |
| 4.A.2.a.1 | Judging the Qualities of Objects, Services, or People | Assessing the value, importance, or quality of things or people. |
| 4.A.2.a.2 | Processing Information | Compiling, coding, categorizing, calculating, tabulating, auditing, or verifying information or data. |
| 4.A.2.a.3 | Evaluating Information to Determine Compliance with Standards | Using relevant information and individual judgment to determine whether events or processes comply with laws, regulations, or standards. |
| 4.A.2.a.4 | Analyzing Data or Information | Identifying the underlying principles, reasons, or facts of information by breaking down information or data into separate parts. |
| 4.A.2.b | Reasoning and Decision Making | What decisions are made and problems solved in performing this job? |
| 4.A.2.b.1 | Making Decisions and Solving Problems | Analyzing information and evaluating results to choose the best solution and solve problems. |
| 4.A.2.b.2 | Thinking Creatively | Developing, designing, or creating new applications, ideas, relationships, systems, or products, including artistic contributions. |
| 4.A.2.b.3 | Updating and Using Relevant Knowledge | Keeping up-to-date technically and applying new knowledge to your job. |
| 4.A.2.b.4 | Developing Objectives and Strategies | Establishing long-range objectives and specifying the strategies and actions to achieve them. |
| 4.A.2.b.5 | Scheduling Work and Activities | Scheduling events, programs, and activities, as well as the work of others. |
| 4.A.2.b.6 | Organizing, Planning, and Prioritizing Work | Developing specific goals and plans to prioritize, organize, and accomplish your work. |

Supplementary Table 2. Ranges and cutoff points for occupational mental demands indices.

|  | Score range | Low | Medium | High |
| --- | --- | --- | --- | --- |
| **Worker-oriented mental demands**  **Cognitive Abilities** | 23.3 – 58.4 | - 1. - 34.9 | 35 – 46.6 | 46.7 – 58.4 |
| **Job-oriented mental demands**  **Mental Processes** | 29.2 – 76 | 29.2 – 44.7 | 44.8 – 60.3 | 60.4 – 76 |

Supplementary Table 3. Generalized linear model analysis on the association of educational attainment only, and including occupational mental demands on cognitive functioning.

|  | Education only |  | Worker-oriented mental demands |  | Job-oriented mental demands |
| --- | --- | --- | --- | --- | --- |
|  | Coefficient [95 CI] |  | Coefficient [95 CI] |  | Coefficient [95 CI] |
| Medium education | 0.46 [0.43, 0.48] |  | 0.45 [0.43, 0.48] |  | 0.46 [0.43, 0.48] |
| High education | 0.82 [0.79, 0.85] |  | 0.78 [0.76, 0.81] |  | 0.79 [0.76, 0.82] |
|  |  |  |  |  |  |
| Medium occupational mental demands |  |  | 0.09 [0.07, 0.11] |  | 0.06 [0.04, 0.08] |
| High occupational mental demands |  |  | 0.16 [0.12, 0.21] |  | 0.17 [0.13, 0.21] |

These models include age, sex, locality size, hypertension, diabetes, heart attack, stroke, depression and health insurance as controls.

Supplementary Table 4. Generalized linear model analysis on the association of occupational mental demands and cognitive functioning by education level.

|  | Education | | | | |
| --- | --- | --- | --- | --- | --- |
|  | Low |  | Medium |  | High |
|  | Coefficient [95 CI] |  | Coefficient [95 CI] |  | Coefficient [95 CI] |
| Medium cognitive abilities | 0.01 [-0.05, 0.07] |  | 0.05 [0.03, 0.08] |  | 0.14 [0.11, 0.17] |
| High cognitive abilities | 0.34 [0.01, 0.67] |  | 0.20 [0.11, 0.3] |  | 0.20 [0.16, 0.25] |
|  |  |  |  |  |  |
| Medium mental processes | -0.02 [-0.08, 0.04] |  | 0.01 [-0.02, 0.4] |  | 0.09 [0.06, 0.11] |
| High mental processes | 0.08 [-0.19, 0.34] |  | 0.19 [0.09, 0.29] |  | 0.20 [0.16, 0.23] |

Worker-oriented Mental Demands = Cognitive abilities; Job-oriented Mental Demands = Mental processes.

These models include age and sex as control variables.


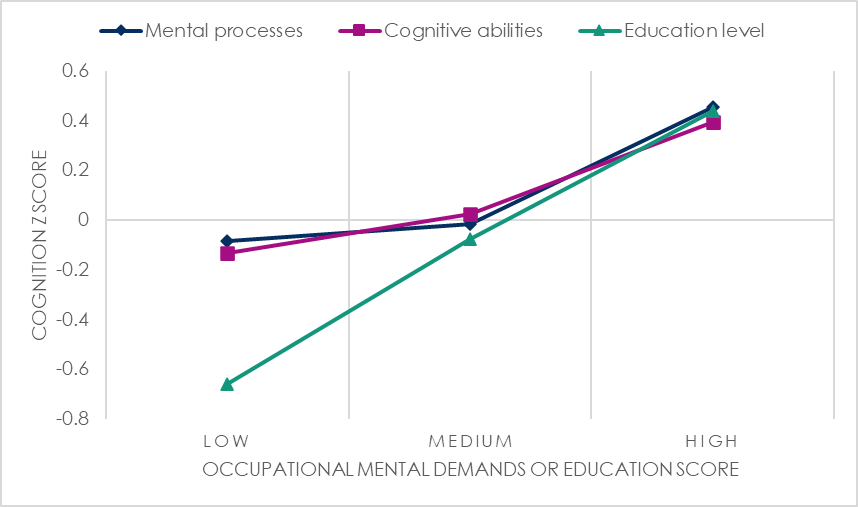


Supplementary Figure 1. Cognition average Z score by occupational mental demands and education. Cognitive abilities = worker-oriented mental demands; Mental processes = job-oriented mental demands.

Supplementary Figure 2. Distribution of participants by level of occupational mental demands and educational attainment stratified by job category according to the INEGI job classification. Each bar represents the percentage of participants in a job category, segmented into low, medium, and high. Cognitive abilities = worker-oriented mental demands; Mental processes = job-oriented mental demands.
